# Angiotensin II Upregulates Endothelial Lipase Expression via the NF-Kappa B and MAPK Signaling Pathways

**DOI:** 10.1371/journal.pone.0107634

**Published:** 2014-09-24

**Authors:** Xiaoli Zhang, Minghui Wu, Hong Jiang, Jing Hao, Qingli Zhang, Qing Zhu, Gaowa Saren, Yun Zhang, Xiaohui Meng, Xin Yue

**Affiliations:** 1 Key Laboratory of Cardiovascular Remodeling and Function Research, Chinese Ministry of Education and Chinese Ministry of Public Health, Department of Cardiology, Qilu Hospital, Jinan, China; 2 Key Laboratory of the Ministry of Education for Experimental Teratology, Department of Histology and Embryology, School of Medicine, Shandong University, Jinan, China; 3 Department of Morphology Laboratory, School of Medicine, Shandong University, Jinan, China; 4 Institute of Diagnostics, School of Medicine, Shandong University, Jinan, China; Thomas Jefferson University, United States of America

## Abstract

**Background:**

Angiotensin II (AngII) participates in endothelial damage and inflammation, and accelerates atherosclerosis. Endothelial lipase (EL) is involved in the metabolism and clearance of high density lipoproteins (HDL), the serum levels of which correlate negatively with the onset of cardiovascular diseases including atherosclerosis. However, the relationship between AngII and EL is not yet fully understood. In this study, we investigated the effects of AngII on the expression of EL and the signaling pathways that mediate its effects in human umbilical vein endothelial cells (HUVECs).

**Methods and Findings:**

HUVECs were cultured *in vitro* with different treatments as follows: 1) The control group without any treatment; 2) AngII treatment for 0 h, 4 h, 8 h, 12 h and 24 h; 3) NF-κB activation inhibitor pyrrolidine dithiocarbamate (PDTC) pretreatment for 1 h before AngII treatment; and 4) mitogen-activated protein kinase (MAPK) p38 inhibitor (SB203580) pretreatment for 1 h before AngII treatment. EL levels in each group were detected by immunocytochemical staining and western blotting. HUVECs proliferation was detected by MTT and proliferating cell nuclear antigen (PCNA) immunofluorescence staining. NF-kappa B (NF-κB) p65, MAPK p38, c-Jun N-terminal kinase (JNK), extracellular signal-regulated kinase (ERK) and phosphorylated extracellular signal-regulated kinase (p-ERK) expression levels were assayed by western blotting. The results showed that the protein levels of EL, NF-κB p65, MAPK p38, JNK, and p-ERK protein levels, in addition to the proliferation of HUVECs, were increased by AngII. Both the NF-kB inhibitor (PDTC) and the MAPK p38 inhibitor (SB203580) partially inhibited the effects of AngII on EL expression.

**Conclusion:**

AngII may upregulate EL protein expression via the NF-κB and MAPK signaling pathways.

## Introduction

Activation of the renin-angiotensin system strongly promotes inflammation in the arterial wall, and has been shown to accelerate atherosclerosis in both mice and humans [1]–[4]. AngII is the most well-described and most active component in the renin-angiotensin system. Recent studies have shown that AngII has also non-hemodynamic effects, such as prothrombotic activity. Several studies suggest that AngII influences fibrinolysis [5]–[8], coagulation [9], [10] and platelet activation [11]–[14], which can promote thrombosis. Moreover, Mogielnicki et al. found that AngII may enhance venous thrombus formation in vivo [15]. AngII is also a direct vasoconstrictor, constricting arteries and veins leading to increased blood pressure and contributing to atherosclerosis [16]–[19].

Endothelial lipase (EL), which belongs to the lipase family, is a key enzyme with phospholipase activities that plays very important roles in the metabolism of HDL [20]. The other functions of EL include increasing the uptake of apolipoprotein B by endothelial cells and the adhesion of monocytes and macrophages to endothelial cells [21]. Animal studies have shown that overexpression of EL decreases the atherosclerotic plaque area in apo-E knockout mice [22]. Consequently, EL expression is closely linked with the pathogenesis of atherosclerosis. EL expression is subject to many factors, and can be increased by shear forces that induce inflammation and blood pressure [23], [24]. However, the precise mechanisms that underlie the regulation of EL expression are not fully elucidated.

NF-κB is found in many types of cells and is involved in cellular responses to various stimuli, such as stress, cytokines, oxidized low-density lipoproteins and bacterial or viral antigens [25]–[29]. NF-κB also plays a key role in inflammatory diseases, including atherosclerosis [30].

The MAPK superfamily comprise four subfamilies: C-JunN terminal kinase stress-activated protein kinases (JNKs/SAPKs), ERKs, big mitogen-activated protein kinase I, and MAPK p38. MAPK p38 is involved in directing cellular responses to various stimuli and in the regulation of cellular processes, such as proliferation and differentiation, cell survival and apoptosis [31], [32]. The inhibition of MAPK p38 has been shown to act as a clinical intervention in chronic obstructive pulmonary disease [33].

次Recent studies have shown that the blockade of NF-κB expression can inhibit EL expression, and the authors suggested that EL gene expression may be regulated by NF-κB [34]. The aim of this study was to investigate the effect of AngII on EL expression in HUVECs cultured in vitro and the possible signaling pathways that mediate this effect. Before AngII treatment, HUVECs were pretreated with inhibitors of either NF-κB or MAPK p38 to explore the molecular mechanisms that underlie this process. The protein levels of other MAPK superfamily members, JNK, ERK, and p-ERK, were also detected by western blotting.

## Materials and Methods

### Reagents

AngII, endothelial cell growth factor (EGF) and a protease inhibitor cocktail were purchased from Sigma (St. Louis, MO, USA); anti-JNK rabbit primary antibody, anti-ERK and anti-p-ERK mouse primary antibody, MTT assay kits, the NF-κB inhibitor (PDTC), the MAPK p38 inhibitor SB203580, RIPA Lysis Buffer, BCA Protein Assay kits were purchased from Beyotime (Beijing, China); phosphate buffer solution (PBS), trypsin and M199 media were from Hyclone (Logan, UT, USA); The rabbit polyclonal anti- EL primary antibody was from Cayman Chemicals (Ann Arbor, MI, USA); anti-von Willebrand factor (vWF, also known as factor VIII-related antigen) mouse monoclonal primary antibody was purchased from Abcam Inc. (Cambridge, MA, USA); anti-PCNA mouse primary antibody, anti-MAPK p38 rabbit primary antibody, anti-NF-kB p65 rabbit primary antibody, the rabbit IgG-immunohistochemical SABC kit, the DAB (diaminobenzidine) kit, FITC- and TRITC-conjugated anti-mouse IgG and the monoclonal anti-β-actin mouse primary antibody were purchased from Zhongshanjinqiao Biotechnology (Beijing, China); fetal bovine serum (FBS) was from Yuanpeng Biotech Co. (Jinan, China); penicillin was from North China Pharmaceutical Co. (Shijiazhuang, China); and streptomycin was from Merro Pharmaceutical Co. (Dalian, China). PVDF membranes were from Millipore (Millipore Corp, MA, USA). Electrochemiluminescence kits were from Amersham (Amersham Life Sciences Inc., IL, USA).

### Ethical approval

This study was conducted on the approval of Ethical Committee at the school of Medicine, Shandong University (Permit Number: 200800243). Written informed consent for the donation of the umbilical cords used in this study was obtained from all patients.

### Cell culture

HUVECs were established from a 15-cm length of umbilical cord. Briefly, the umbilical vein was washed inPBS solution for 5 min. Subsequently, 0.25% trypsin-EDTA (0.02%) was injected into the lumen of the umbilical vein and allowed to digest for 10–15 min at room temperature with gentle shaking to allow full contact of the enzyme with the vascular wall. The solution was then collected in a 50 ml tube and centrifuged at 888×*g* for 10 minutes. The supernatant was removed and cells were resuspended at 3×10^5^ cells/ml in 20% FBS cell culture medium (M199 + 20% FBS +100 U/ml penicillin +100 U/ml streptomycin +3 µl/ml EGF). The cell suspension was transferred to a 6-well culture plate (6×10^5^ cells/well) and incubated in a saturated humidity, under 5% CO_2_ in a 37°C incubator. The medium was changed every 2 or 3 days. Cells were passaged at 80–90% confluence and the passaged cells were subsequently incubated in 10% FBS culture media. The purity of HUVECs (at >80% confluence) was determined by the immunofluorescent staining of vWF.

### Cell treatments

Cells were divided into three groups and treated after the third passage. The groups were treated as follows: 1) control group, without any treatment; 2) AngII (10 µmol/L); 3) PDTC (10 mmol/L) pretreatment for 1 h + AngII (10 µmol/L); and 4) SB203580 (10 µmol/L) pretreatment for 1 h + AngII (10 µmol/L). Cells from each group were collected at 0 h, 2 h, 4 h, 8 h, 12 h, and 24 h after the initial treatments for further analysis.

### Immunocytochemical staining

Coverslips coated with cells were washed in PBS. The cells were then fixed in methanol: acetic acid (3∶1) for 10 min and washed in PBS. Then cells were immunoblocked with normal goat serum and permeabilized in 0.01 M PBS containing 0.3% Triton X-100 at RT for 1 h. Cells were incubated with primary antibodies for the detection of EL (1∶100) and PCNA (1∶100) in a humidified box at 4°C overnight. Cells were then incubated with biotin-conjugated anti-rabbit IgG or TRITC-conjugated anti-mouse IgG (for PCNA staining) at 37°C for 1 h. Cells were examined using an Olympus U-LH100HG microscope (for PCNA staining) directly or treated with SABC complex at 37°C for 1 h according to the manufacturer's instructions and immunoreactions were visualized using the DAB kit. Cells were washed twice in PBS between sequential steps of the procedure. Negative control staining was performed with nonspecific IgG instead of the primary antibody. Cells were counterstained with hematoxylin or DAPI and finally sealed with neutral balsam or anti-fade (for PCNA staining) and examined using an Olympus U-LH100HG microscope.

### Western blotting

EL, MAPK p38, NF-kB p65, JNK, ERK and p-ERK protein levels in HUVECs were detected by western blotting. Briefly, HUVECs in each group were harvested separately and washed in cold PBS, and homogenized at 4°C in lysis buffer containing 10 mM Hepes pH 7.9, 10 mM KCl, 1.5 mM MgCl_2_, 0.1 mM EGTA, 0.5 mM DTT, 10 mM β-glycerophosphate, 0.1 mM sodium vanadate and a protease inhibitor cocktail. After 15 min of incubation on ice, cell debris was removed by centrifugation at 15,000×g for 20 min at 4°C. Protein concentration was determined by the BCA assay with BSA as a standard. Proteins were separated on a 10% SDS-polyacrylamide gel, and then transferred to a PVDF membrane. After blocking with 5% (w/v) fat-free milk for 1 h at room temperature, the membranes were probed with primary antibodies overnight at 4°C, followed by incubation with peroxidase-conjugated IgG for 1 h at room temperature. The interaction was monitored with an electrochemiluminescence kit. Detection of β-actin was performed as a loading control.

### MTT assays

The MTT assay is a colorimetric assay for assessing cell viability. It was performed according to the instructions of the manufacturer. Briefly, cells (3.0×10^4^/well) were grown in a 96-well flat-bottomed culture plate at 37°C in a humidified atmosphere with 5% CO_2_. After 96 h, 10 µl of the MTT (final concentration 0.5 mg/ml) was added to each well and the plate was incubated for a further 4 h prior to the addition of 100 µl of formazan to each well. The plate was incubated overnight and the absorbance of the samples at 570 nm was measured using a microtiter plate reader (BIO-RAD Model 680).

### Statistical analysis

Densitometric evaluation of Western blot results was conducted using the Quantity One software with β-actin as an internal control. Data were presented as the mean ± standard deviation (SD) of three separate experiments. Comparisons among groups were conducted using one-way analysis of variance (ANOVA). If the result of the ANOVA was statistically significant, then multiple comparison tests between groups were performed using the Student-Newman-Keuls (SNK) method. Results were considered statistically significant at *P*<0.05.

## Results

### Isolation and identification of HUVECs

**Figure 1:**
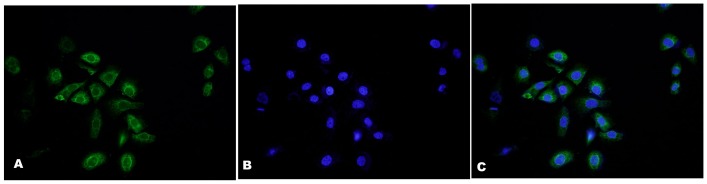
Immunofluorescence staining of vWF was performed to identify HUVECs cultured in vitro. The panels show immunostaining of vWF (A) and DAPI staining of nuclei (B) with merged images (C). Almost all cells (>95%) were vWF-positive.

Immunofluorescence staining of vWF, which is specifically expressed in blood vessel endothelial cells, was performed to identify HUVECs. The results showed that the isolated cells were almost all (>95%) vWF-positive (Fig. 1).

### Addition of AngII increased the EL expression in HUVECs

**Figure 2:**
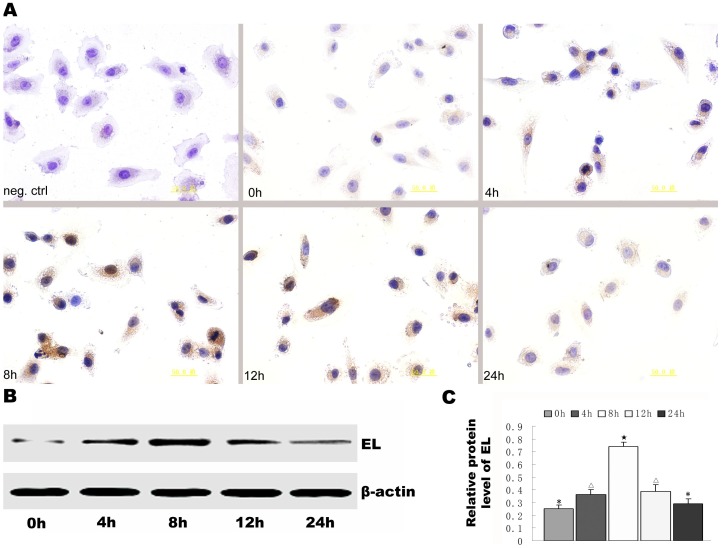
AngII treatment was shown to increase EL expression in HUVECs by immunocytochemical staining and Western blot analyses. (A) Negative control and the immunocytochemical staining of EL at 0 h, 4 h, 8 h, 12 h and 24 h after AngII treatment. (B) EL expression detected by western blotting at 0 h, 4 h, 8 h, 12 h and 24 h after AngII treatment. (C) Semi-quantitative analysis of the EL levels detected by western blotting. Different symbols represent statistical significance (*p*<0.05).

After HUVECs were stimulated with AngII, EL expression levels in different groups were assayed by immunocytochemical staining and western blotting. The results showed that EL levels increased significantly from 4 h after AngII treatment. The EL levels persisted at significantly (*p*<0.05) high levels until 12 h after treatment and then decreased to almost baseline levels at 24 h after treatment (Fig. 2).

### Addition of AngII promoted proliferation of HUVECs

**Figure 3:**
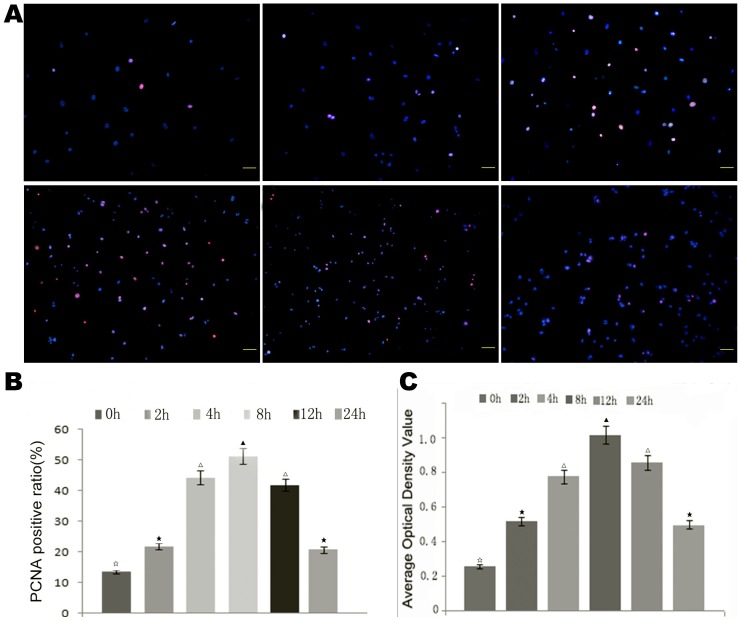
AngII treatment promoted proliferation of HUVECs. AngII treatment was shown to promote HUVEC proliferation by immunofluorescence staining of PCNA and MTT assays. (A) Immunofluorescence staining of PCNA of HUVECs in each group. (B) Semi-quantitative analysis of the immunofluorescence staining of PCNA. (C) Shows the statistical analysis of MTT assays for each group. Different symbols indicate statistical significance (*p*<0.05).

In order to detect the effects of increased EL on HUVECs, immunofluorescent staining of PCNA and MTT was performed to detect the proliferative activity of HUVECs at 0 h, 2 h, 4 h, 8 h, 12 h, and 24 h. The results showed that increased EL promoted HUVEC proliferation (Fig. 3).

### Addition of AngII increased MAPK p38 and NF-κB p65 expression in HUVECs

**Figure 4:**
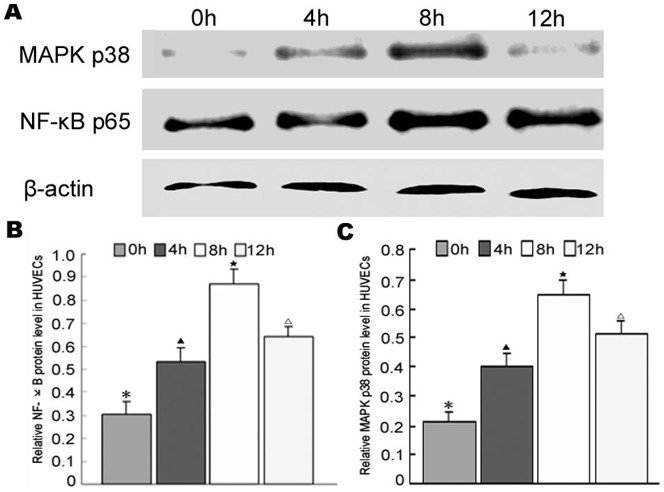
AngII treatment was shown to increase NF-κB p65 and MAPK p38 expression in HUVECs by western blotting. (A) MAPK p38 and NF-κB p65 expression levels detected by western blotting. (B) Semi-quantitative analysis of the NF-κB levels detected by western blotting. (C) Semi-quantitative analysis of the MAPK p38 levels detected by western blotting. Different symbols indicate statistical significance (*p*<0.05).

To investigate the possible signaling pathways that mediate the function of AngII, expression levels of MAPK p38 and NF-κB p65 were detected by western blotting at 0 h, 4 h, 8 h and 12 h. The results showed that both MAPK p38 and NF-κB p65 expression levels increased significantly at 4 h, 8 h, and 12 h compared to those detected at 0 h (Fig. 4).

### NF-κB inhibitor PDTC or MAPK p38 inhibitor SB203580 inhibited the EL increase induced by AngII in HUVECs

**Figure 5:**
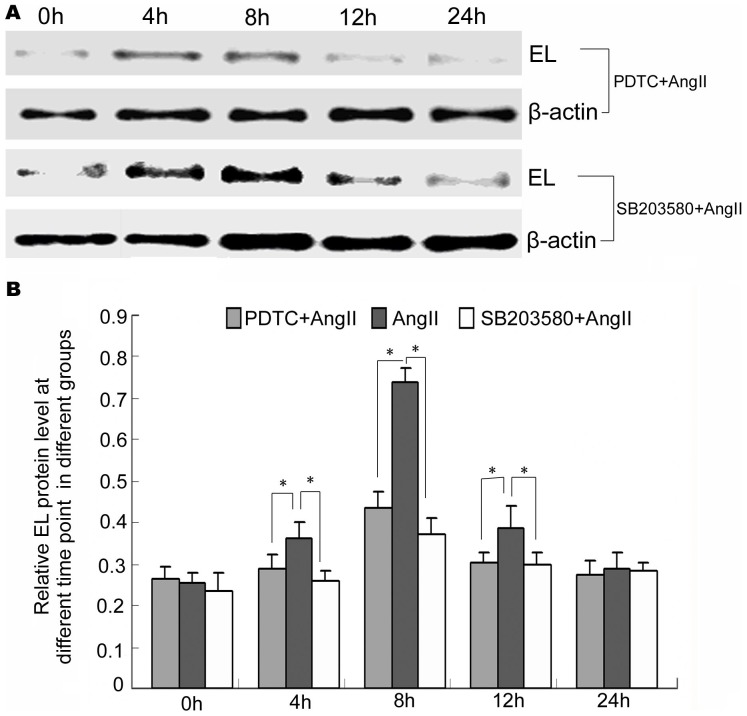
Blockade of NF-κB p65 or MAPK p38 was shown to decrease EL levels in HUVECs by western blotting. (A) EL expression levels in different groups detected by western blotting. (B) Semi-quantitative analysis of the EL levels detected by western blotting. * Indicates statistical significance (*p*<0.05).

In order to determine if the increase of EL in HUVECs induced by AngII is mediated by MAPK p38 or NF-κB p65, HUVECs cultured in vitro were pretreated with inhibitors of MAPK p38 or NF-κB for 1 h before AngII treatment. Cells were harvested at 0 h, 4 h, 8 h, 12 h and 24 h after AngII treatment and EL expression levels were detected by western blotting. The results showed a significant reduction in EL levels at 4 h, 8 h and 12 h in the PDTC + AngII and SB203580 + AngII groups compared to that detected in the AngII treated group (Fig. 5).

### Addition of AngII increased JNK and p-ERK expression in HUVECs

**Figure 6:**
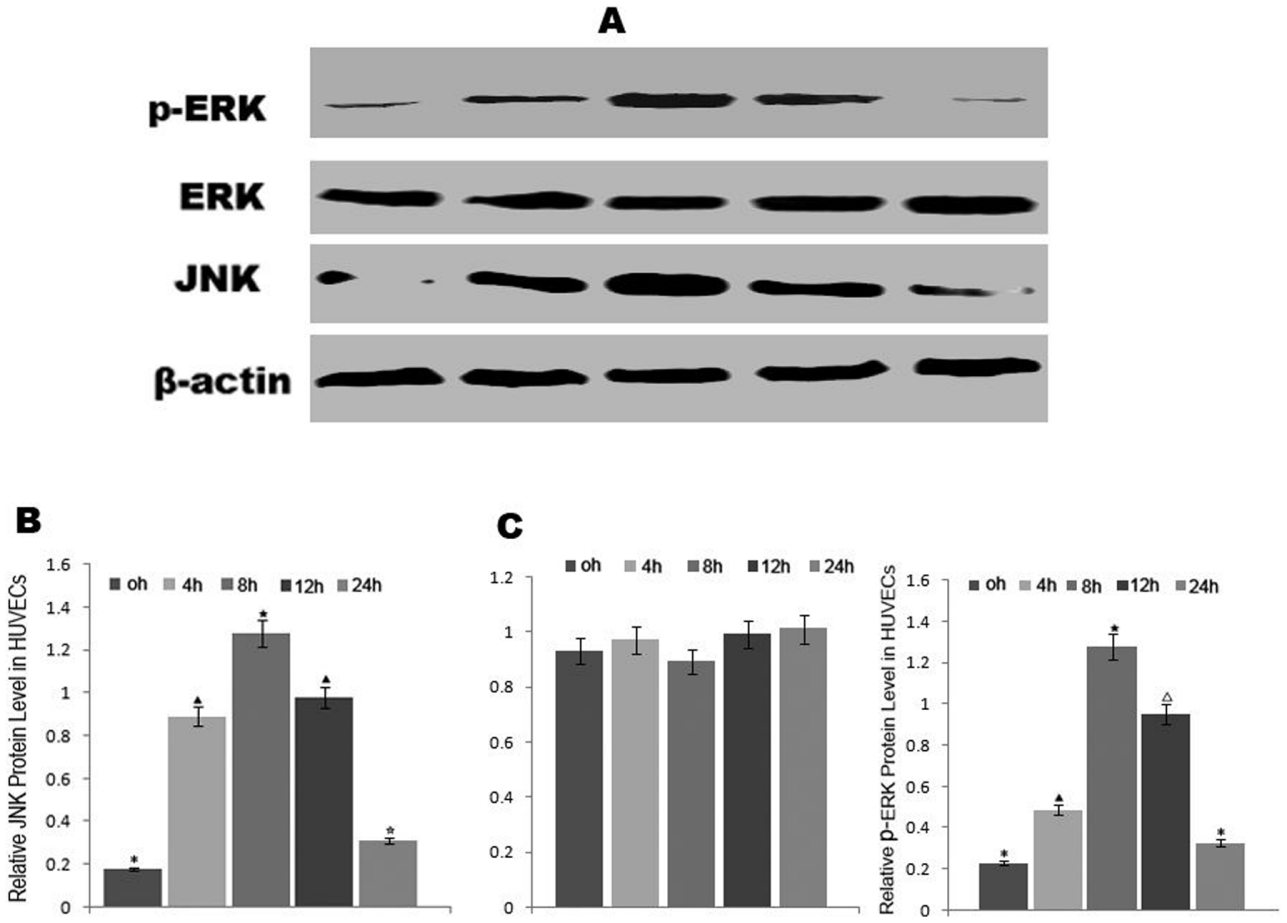
AngII treatment increased JNK and P-ERK expression in HUVECs by western blotting. (A) JNK and P-ERK expression levels. (B) Semi-quantitative analysis of the JNK levels. (C) Semi-quantitative analysis of the levels. Different symbols indicate statistical significance (*p*<0.05). The same symbols or no symbols indicate no statistical significance (*p*>0.05).

To investigate whether JNK and ERK were involved in the increased expression of EL regulated by AngII, expression levels of JNK, ERK and p-ERK were detected by western blotting at 0 h, 4 h, 8 h, 12 h, and 24 h. The results showed that both JNK and p-ERK expression levels increased significantly at 4 h, 8 h, and 12 h compared to those detected at 0 h, ERK protein levels did not change significantly (Fig. 6).

## Discussion

EL was discovered in 1999 as part of the triglyceride lipase family of genes [20]. EL has an essential phospholipase activity and is a critical enzyme in HDL metabolism [35]. The plasma HDL concentration is significantly increased in EL gene knockout mice and EL overexpression markedly reduces HDL plasma levels, which is an independent risk factor for atherosclerosis [36]. Fang et al. found increased EL expression in vascular endothelial cells of patients with coronary heart disease, and this increase was shown to correlate with the severity of the clinical syndrome and the increasing coronary risk scores[37]. Furthermore, polymorphisms of the gene that encodes EL may be related to the progression of acute coronary syndrome [38], [39].

Atherosclerosis is a chronic inflammatory disease, characterized by the interaction of inflammatory mediators and cytokines with the vascular endothelium. In 2000, the first report was published showing that interleukin-1β and tumor necrosis factor alpha (TNFα) upregulate the mRNA expression of EL in HUVECs in vitro [24]. Jin et al. [23] confirmed that this finding was partially mediated through the NF-κB pathway. Badellino et al. [40] and Paradis et al. [41] found that high-sensitivity C-reactive protein, soluble tumor necrosis factor-receptor, soluble intracellular adhesion molecule, IL-6 and other cytokines are positively correlated with EL levels. Additionally, vascular pressure and shear forces on vascular walls increase the risk of atherosclerosis, decrease levels of HDL and increase the transcription of EL [42]. In the current study, our results showed that AngII stimulation increased EL expression between 4 h and 12 h after treatment, with the highest level at 8 h. Thus, our results further confirmed the positive correlation between AngII and EL levels in HUVECs cultured in vitro as well as the time-dependent effects of AngII on EL expression. Increased EL promoted the proliferative activity of HUVECs and may contribute to the pathogenesis of atherosclerosis, which is in accordance with our previous report [43].

NF-κB is an important transcriptional regulatory factor. When cells are stimulated by various cytokines, NF-κB is activated and enters the nucleus where it combines with specific DNA motifs and stimulates the expression of various genes. Physical factors such as shear stress can also activate NF-κB and then promote secretory functions of vascular endothelial cells [44]. Chromatin immunoprecipitation and electrophoretic mobility shift assays have revealed that the EL gene has five NF-κB binding sites [45]. In this study, endothelial cells were pretreated with an inhibitor of NF-κB (PDTC) and were then stimulated by AngII. EL expression was significantly reduced, which suggested that EL expression is regulated by AngII through the NF-κB signal transduction pathway.

The MAPKs constitute the components of a cascade of reactions that are some of the most important intracellular signal transduction pathways. They respond to a wide range of extracellular stimuli and have been associated with endothelial dysfunction, inflammation, hypertension and vascular remodeling [46]–[49]. The MAPK superfamily comprise four subfamilies: JNKs/SAPKs, ERKs, big mitogen-activated protein kinase I and MAPK p38. Paravicini et al. [50] determined that AngII stimulation induces the expression of procollagenase I in rat smooth muscle cells and accelerates vascular fibrosis. The development of fibrosis can be ameliorated by the inhibition of p38 MAPK by SB203580, which suggests that the signal is transmitted via the p38 MAPK pathway. SB203580 inhibits MAPK p38 selectively and has no significant inhibitory effect on JNK/SAPK or ERK. Our current study showed that the pretreatment of HUVECs with SB203580 also inhibited the effects of AngII in promoting the expression of EL.

To investigate whether the JNK or ERK signaling pathways were also involved in the increased expression of EL regulated by AngII, the protein level of JNK and p-ERK were also detected by western blotting. Chen Huan previously reported that AngII treatment can increase the mRNA level of JNK in HUVECs [51]. Jun et al. reported that AngII induces p-ERK [52]. Our results were in accordance with these previous findings.

Our findings therefore confirm that AngII regulates the expression of EL via the NF-κB and MAPK signaling pathways. This preliminary in vitro study suggests that both the NF-kB and MAPK signaling pathways participate in the regulation of EL expression and may therefore be linked to atherosclerotic risk. Although these results cannot be extrapolated to in vivo situations, this study provides evidence of a possible mechanism by which AngII affects the EL expression in endothelial cells. We hope that our study will provide the theoretical and experimental basis for future preventative treatments that specifically target these factors.
