# Correction: Angiotensin II Upregulates Endothelial Lipase Expression via the NF-Kappa B and MAPK Signaling Pathways

**DOI:** 10.1371/journal.pone.0118768

**Published:** 2015-03-05

**Authors:** The PLOS ONE Staff

In the author affiliations, affiliation 1 and affiliation 2 are erroneously switched. Affiliation 1 should be: Key Laboratory of the Ministry of Education for Experimental Teratology, Department of Histology and Embryology, School of Medicine, Shandong University, Jinan, China. Affiliation 2 should be: Key Laboratory of Cardiovascular Remodeling and Function Research, Chinese Ministry of Education and Chinese Ministry of Public Health, Department of Cardiology, Qilu Hospital, Jinan, China. Please view the correct author affiliations here:

Xiaoli Zhang^1^, Minghui Wu^2^, Hong Jiang^2^, Jing Hao^1^, Qingli Zhang^3^, Qing Zhu^2^, Gaowa Saren^2^, Yun Zhang^2^, Xiaohui Meng^4^, Xin Yue^2^*

1. Key Laboratory of the Ministry of Education for Experimental Teratology, Department of Histology and Embryology, School of Medicine, Shandong University, Jinan, China.

2. Key Laboratory of Cardiovascular Remodeling and Function Research, Chinese Ministry of Education and Chinese Ministry of Public Health, Department of Cardiology, Qilu Hospital, Jinan, China.

3. Department of Morphology Laboratory, School of Medicine, Shandong University, Jinan, China.

4. Institute of Diagnostics, School of Medicine, Shandong University, Jinan, China.
